# Change and variability in drug treatment coverage among people who inject drugs in 90 large metropolitan areas in the USA, 1993–2007

**DOI:** 10.1186/s13011-018-0165-2

**Published:** 2018-08-09

**Authors:** Barbara Tempalski, Charles M. Cleland, Leslie D. Williams, Hannah L. F. Cooper, Samuel R. Friedman

**Affiliations:** 1 Institute for Infectious Disease Research, NDRI, Inc., 71 West 23rd Street, 4th Floor, New York, NY 10010 USA; 2 New York University College of Nursing, New York, NY USA; 3 Rollins School of Public Health, Emory University, Atlanta, GA USA; 4 Department of Epidemiology, Bloomberg School of Public Health, Johns Hopkins University, Baltimore, MD USA

**Keywords:** People who inject drugs, Opioids, Drug treatment coverage, Change over time, Metropolitan statistical areas

## Abstract

**Background:**

Our previous research has found low and stable mean drug treatment coverage among people who inject drugs (PWID) across 90 large US metropolitan statistical areas (MSAs) during 1993–2002. This manuscript updates previous estimates of change in drug treatment coverage for PWID in 90 MSAs during 1993–2007.

**Methods:**

Our drug treatment sample for calculating treatment coverage includes clients enrolled in residential or ambulatory inpatient/outpatient care, detoxification services, and methadone maintenance therapy at publicly- and privately-funded substance abuse agencies receiving public funds. Coverage was measured as the number of PWID in drug treatment, calculated by using data from the Substance Abuse and Mental Health Service Administration, divided by numbers of PWID in each MSA. We modeled change in drug treatment coverage rates using a negative binomial mixed-effects model. Fixed-effects included an intercept and a main effect for time. Incidence rate ratios (IRR) were calculated for both average change from 1993 to 2007 and MSA-specific estimates of change in coverage rates.

**Results:**

On average over all MSAs, coverage was low in 1993 (6.1%) and showed no improvement from 1993 to 2007 (IRR = 0.99; 95% CI, 0.86, 1.2). There was modest variability across MSAs in coverage in 1993 (log incidence rate SD = 0.36) as well as in coverage change from 1993 to 2007 (log IRR SD = 0.32). In addition, results indicate significant variability among MSAs in coverage.

**Conclusions:**

Inadequate treatment coverage for PWID may produce a high cost to society in terms of the spread of overdose mortality and injection-related infectious diseases. A greater investment in treatment will likely be needed to have a substantial and more consistent impact on injection drug use-related harms. Future research should examine MSA-level predictors associated with variability in drug treatment coverage.

## Introduction

In the United States, the misuse of and addiction to opioids – including prescription opioid analgesics, heroin, and synthetic opioids – is a serious national problem that affects public health as well as social and economic welfare [1–5]. In 2015, over 33,000 Americans died as a result of an opioid-related overdose, another 2.5 million people suffered from substance use disorders related to prescription opioid analgesics, heroin, and synthetic opioids [4, 6–10]. Meanwhile, the cost of prescription opioids, combined with their shrinking availability, has led opioid users to the cheaper and more readily available alternative of heroin [4, 11–14]. This trend has led to a wave of new young heroin injectors, escalating the risk of HIV and HCV and drug-related mortality among this cohort [12, 13, 15–18]. Given the magnitude of this ongoing public health epidemic, it is critical to understand the availability and coverage of drug treatment services for those in need of them.

### Significance of understanding drug treatment coverage rates among PWID

Drug treatment for PWID is effective in reducing harms and improving users’ health outcomes [19–23]. Evidence-based drug treatment, such as methadone maintenance therapy and buprenorphine, can also address a broad range of social and public health problems [17, 19, 21, 23–25], especially when programs provide access to AIDS education and prevention programs, HIV and HCV testing, and contact with health care systems to those clients already infected with HIV [20–23, 25]. Adequate access and expansion to effective treatment and medication assisted therapies for opioid dependence has led to improved antiretroviral therapy (ART) adherence and decreases in morbidity among PWID [17, 25–32].

Additionally, research on the effectiveness of drug treatment indicates that increased length of time in treatment is associated with lower rates of needle sharing and HIV seropositivity [18, 23, 25, 27–30]. Treatment may also be related to reductions in overdose, illicit drug use, and unemployment among drug users in general, although these effects may vary by treatment modality and access to treatment service facilities [5, 23, 26, 31, 32]. In view of such evidence, public health would clearly benefit if drug treatment were widely available to drug injectors.

Prior research on treatment coverage among PWID estimated the number of injectors and the extent of treatment coverage in 90 US metropolitan statistical areas (MSAs) for 1993–2002, reporting that drug treatment coverage was low in 1993 (mean 6.7%; median 6.0%) and decreased slightly in 2002 (mean of 8.3%; median of 8.0%) [33]. Here we present a brief update on drug treatment coverage rates among PWID from 1993 to 2007 in 90 MSAs in the USA.

## Methods and data

### Overview

We define treatment coverage as the ratio of PWID in treatment to total PWID in each MSA. Percentage of PWID in treatment for each year from 1993 to 2007 (excluding years 1994, 1999, and 2001) was calculated using data on drug treatment entries and reported injection at drug treatment intake from the Substance Abuse and Mental Health Service Administration (SAMHSA) [34–37], and population estimates of PWID from our previous research [38].

#### Unit of analysis and sample

As previously noted in Tempalski et al. (2010) [33], the unit of analysis in this study is the MSA. The US Census Bureau and Office of Management and Budget define an MSA as a set of contiguous counties that include one or more central cities of at least 50,000 people that collectively form a single, cohesive socioeconomic unit, defined by inter-county commuting patterns and socioeconomic integration [39]. MSAs are meaningful epidemiologic units within which to study PWID and services designated for them [40].

#### Data

We calculated treatment coverage rates using two data series from the SAMHSA [34–37], and estimates of PWID from previous research estimates [38]. Table 1 describes each database utilized in calculating drug treatment coverage rates.

**Table 1:** Description of Data Sources Utilized to Calculate Drug Treatment Coverage Rates^a^

1) Proportion of treatment entrants who indicated that they injected substances intravenously in each MSA and year (1993–2007) as reported by the Treatment Episode Data Set (TEDS) [34]; 2) Total number of drug users in drug treatment as of October 1 of each year reported by the Uniform Facility Data Set (UFDS) for 1993, 1995, 1996–1998 [35, 36] and the National Survey of Substance Abuse Treatment Services (N-SSATS) for 2000, 2002–2007 [37]; 3) Total estimated number of PWID in each MSA and year (1993–2007) as calculated and reported by Tempalski and colleagues [38]^b^	

^a^These data do not capture medication-assisted treatment (MAT) operating out of private medical offices. Additionally, we need to acknowledge much of any system response to the opioid epidemic would have taken place after 2007, and so our data would miss those more recent changes

^b^Our drug treatment coverage estimates are based on the number of PWID in an MSA. Current data available for PWID are up through 2007

#### Calculating number of PWID

Tempalski and colleagues [38] first estimated the number of PWID in the US each year from 1992 to 2007 and then apportioned these estimates to MSAs using multiplier methods. Four different types of data indicating drug injection were used to allocate national annual totals to MSAs, creating four distinct series of estimates of the number of injectors in each MSA. These estimates rely on using (1) HIV counseling and testing data from the Centers for Disease Control (CDC) [41]; (2) SAMSHA’s UFDS and TEDS data [34–37]; (3) CDC’s diagnoses of PWIDs with HIV/AIDS [42]; and (4) an estimate derived from published estimates of the number of injectors living in each MSA in 1992 [43] and in 1998 [44]. Each series was smoothed over time using loess regression and the mean value of the four component estimates was taken as the best estimate of PWID for that MSA and year. In order to avoid circularity, the estimated numbers of PWID in the population used in this study modify the Tempalski estimates [38] so that they do not rely on data on the numbers of PWID in drug treatment from SAMSHA.

#### Calculating drug treatment coverage rates

We define treatment coverage as the ratio of PWID in treatment to PWID in the MSA. Our drug treatment sample for calculating treatment coverage includes clients enrolled in residential or ambulatory inpatient/outpatient care, detoxification services, and methadone maintenance therapy at publicly- and privately-funded substance abuse agencies receiving public funds. These are facilities licensed, certified, or otherwise approved by State substance abuse agencies to provide substance abuse treatment. Buprenorphine patients are not included in these data unless their buprenorphine is provided by one of these agencies.

Treatment coverage for PWID is estimated using TEDS and UFDS/N-SSATS. Here, the difficulty is that neither data set provides an estimate of the number of clients who inject drugs. UFDS/N-SSATS does provide data on the number of clients in these treatment services. We adjust these estimates by multiplying them by the proportion of treatment *entrants* who inject drugs, which can be calculated from TEDS data. The following equation calculates drug treatment coverage rates:

Ajt = treatment one-day census total (Djt) adjusted for proportion of these who inject drugs (Bjt / Cjt) divided by Tempalski estimates of number of PWID in the MSA in year t (Ejt), expressed as a percentage =100* (Bjt /(Djt * (Bjt / Cjt)) / Ejt

where,

*A*_*jt*_ = treatment coverage rate for an MSA *j* in year *t*;

*B*_*jt*_ = number of PWID entering drug treatment as reported by TEDS for an MSA *j* in year *t*;

*C*_*jt*_ = number of PWID and number of non-injectors^1^ entering drug treatment as reported by TEDS for an MSA *j* in year *t*;

*D*_*jt*_ = number of drug users in drug treatment reported by UFDS/N-SSATS for an MSA *j* in year *t*; and.

*E*_*jt*_ = estimated number of PWID as estimated by Tempalski et al. [38] for an MSA *j* in year *t*.

First, the TEDS data series identifies the number and attributes of clients who enter substance abuse treatment programs that receive any state and federal funding. From TEDS, we calculated the proportion of treatment entrants who reported they injected drugs as a mode of administration. Our second SAMHSA data source comes from the annual census of drug treatment facilities originally referred to as the Uniform Facility Data Set (UFDS) – but since renamed the National Survey of Substance Abuse Treatment Services (N-SSATS). UFDS/N-SSATS data measure client characteristics and use of privately- and publicly-funded substance abuse treatment programs in the U.S. on October 1 for each year. However, UFDS/N-SSATS data were unavailable for 1992, 1994, 1999, and 2001. As a result of this limited availability, our coverage estimates were only created for years where data were available. Thus, our final drug treatment coverage estimates only provide data for 1993, 1995, 1996–1998, 2000, and 2002–2007.

#### Negative binomial mixed model for estimating change in drug treatment coverage rates

We modeled change in drug treatment coverage rates using a negative binomial mixed-effects model. Fixed-effects included an intercept and a main effect for time. Time was coded such that the intercept captured the coverage rate in 1993, and the time main effect captured the increase in coverage rate from 1993 to 2007. The natural logarithm of PWID population size in each MSA was included as an offset. Both the intercept and the time main effect were allowed to vary randomly across MSAs, yielding MSA-specific estimates of change in addition to average change over all MSAs. Incidence rate ratios (IRR) were calculated for both average change from 1993 to 2007 and MSA-specific estimates of change in coverage rates. The model was fit using PROC GLIMMIX of SAS (Version 9.3). [45]

## Results

### Descriptive statistics

**Fig. 1:**
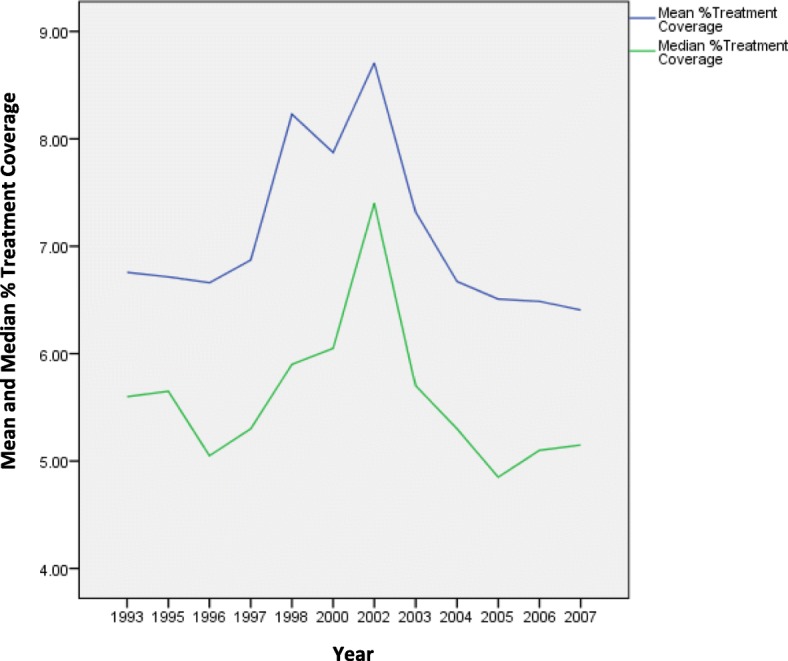
Mean and Median Estimates of Drug Treatment Coverage Rates, 1993-2007

As depicted in Table 2 and in Fig. 1, coverage overall changed little from 1993 to 2007. Mean drug treatment coverage was only 6.4% (standard deviation 4.5%; interquartile range 3.2%-8.9) in 2007, reflecting a slight decrease from 6.7% in 1993 (standard deviation 3.7%; interquartile range 4.2–9.4%). Median treatment coverage decreased from 5.6% in 1993 to 5.2% in 2007.

**Table 2:** Descriptive statistics of estimated PWID drug treatment coverage rates

	Mean	SD	Min	Q_25_	Median	Q_75_	Max
Treatment Coverage^a^	%	%	%	%	%	%	%
1993	6.75	3.70	0.80	4.20	5.60	9.40	16.40
1995	6.71	4.16	0.90	3.60	5.65	9.70	20.60
1996	6.66	4.01	0.90	3.40	5.05	9.70	18.10
1997	6.87	5.47	1.10	3.00	5.30	9.70	28.90
1998	8.22	6.42	0.60	3.40	5.90	12.00	36.00
2000	7.87	5.81	0.50	3.40	6.05	10.30	31.80
2002	8.70	5.38	1.00	4.50	7.40	10.90	26.80
2003	7.31	5.36	0.90	3.20	5.70	9.20	27.30
2004	6.67	5.15	0.90	3.20	5.30	8.10	29.40
2005	6.50	4.99	1.00	2.80	4.85	8.30	22.70
2006	6.48	4.48	0.90	2.90	5.10	8.20	22.30
2007	6.40	4.51	0.90	3.20	5.15	8.90	19.70

^a^Percent in treatment

### Incidence rate ratios of change

Table 3 shows the fixed-effect of time estimates in coverage change from 1993 to 2007 across MSAs indicated a stable rate of overall drug treatment coverage over time (IRR: 0.99, 95% CI: 0.86, 1.25).

**Table 3:** Fixed-Effects Estimates (95% CI)

Average Coverage in 1993	6.1% (5.4, 7.0%)
Rate Change from 1993 to 2007 (IRR)	0.99 (0.86, 1.25)

**Fig. 2:**
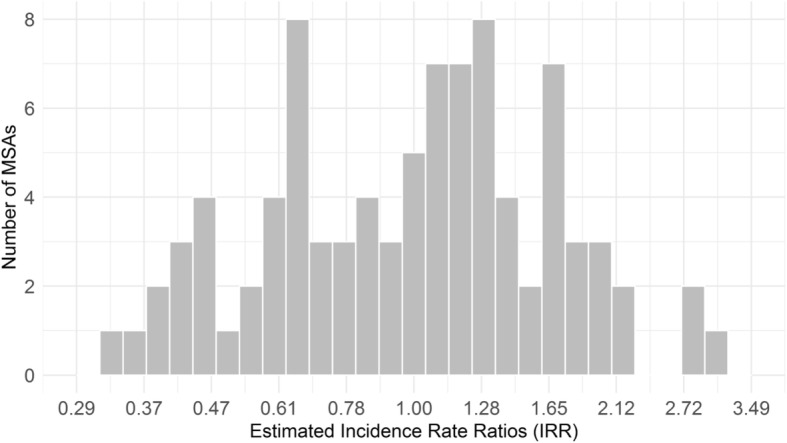
MSA-specific Incidence Rate Ratios Estimates of Change in Drug Treatment Coverage Rates

While the average change in rate over the expanded study period is close to zero, there is substantial variation in rate of change across MSAs. Estimated from the mixed-effects negative binomial model, the standard deviation of log incidence rate ratios was SD = 0.32. MSA-specific IRR estimates of change in coverage rates, which also convey this variability in coverage change over MSAs, are displayed in Fig. 2.

Forty-eight (53%) of the MSAs had an increase (IRR > 1.0) in treatment coverage across the 1993–2007 period. Of those, only eighteen were statistically significant at *p* < =0.05 (Albany-Schenectady, NY; Birmingham, AL; Charlotte-Gastonia-Rock-Hill, NC-SC; Cincinnati, OH-KY-IN; Dayton-Springfield, OH; Detroit, MI; Greenville-Spartanburg, SC; Jersey City, NJ; Knoxville, TN; Louisville, KY-IN; Monmouth-Ocean, NJ; New York, NY; Newark, NJ; Pittsburgh, PA; Raleigh-Durban-Chapel Hill, NC; Richmond-Petersburg, VA; Rochester, NY; Wilmington-Newark, DE-MD).

In addition, forty-one (45%) of the MSAs had a decrease (IRR < 1.0) in treatment coverage across the study period. Eighteen MSAs exhibited a statistically significant decrease in coverage (Bakersfield, CA; Charleston-North Charleston, SC; Dallas, TX; Fresno, CA; Honolulu HI; Houston, TX; Los Angeles-Long Beach, CA; Oakland, CA; Riverside-San Bernardino, CA; San Francisco, CA; San Diego, CA; Stockton-Lodi, CA; Sacramento, CA; Tacoma, WA; Toledo, OH; Tulsa, OK; Youngstown-Warren, OH).

## Discussion

Drug treatment services in the US have largely operated as an independent part of the overall health care system, with unique methods of funding and service delivery. The variability in funding and service delivery varies greatly among US states and even among cities within the same state [46, 47]. Meanwhile, US conservative policies and attitudes toward embracing alternative approaches to treatment, such as harm reduction and opioid agonist therapy, lags well behind much of Western Europe and other regions [33, 48]. As a result, alternative treatments for opioid use disorder such as opioid agonist therapy (i.e., methadone and/or buprenorphine) have not increased to match demand. Such factors highlight the heterogeneous situation in the US with respect to treatment coverage and the fact that treatment provision remains insufficient in so many areas in the US [46, 47, 49, 50].

As previously reported by Tempalski and colleagues [33, 40], treatment coverage for PWID in US MSAs was far below international standards. Some European Union countries, for example, maintain coverage levels of 65% or higher (i.e., France 80%) are in comparison to US coverage rates [48].

Our current estimates show that the average coverage rate change was essentially zero (IRR: 0.99), suggesting no effective, systematic, or country-wide expansion of treatment coverage over the study period. There was however significant variation in coverage rates and coverage rate changes among MSAs. Such variation may be explained by MSA-level factors we have yet to investigate.

Low availability of drug treatment coverage has many public health implications for the US opioid epidemic. In recent years, nonmedical use of prescription opioids has not only been increasing [1–7], but has also been found to be a significant risk factor for heroin use (in particular for injection-related use), unintentional opioid-related overdose, and transmission of HIV and HCV [7–9, 11–14, 16–18], underscoring the need for expanding prevention and drug treatment efforts. Drug treatment coverage will need to increase in order to adequately address need and to curb the recent rise in morbidity and mortality associated with the rise in the misuse of and addiction to opioids.

## Conclusion

Our findings highlight the lack of increase in the ratio between the availability of PWID treatment services and the number of PWID who want or could benefit from drug abuse treatment. Given that drug treatment is effective in reducing harms, a greater investment in drug treatment and the broader services offered at treatment centers must be made to contribute to reducing injection drug use and associated harms. Future research should examine what policy and structural changes affect variations and changes in treatment coverage - and, in particular, what combinations of MSA-level factors lead to increases in treatment coverage.

## Abbreviations

AIDS: Acquired immune deficiency syndrome
AL: Alabama
ART: Antiretroviral therapy
CA: California
CDC: Centers for Disease Control
HCV: Hepatitis C virus
HIV: Human immunodeficiency virus
KY-IN: Kentucky-Indiana
MI: Michigan
MSAs: US metropolitan statistical areas
NC: North Carolina
NJ: New Jersey
N-SSATS: National Survey of Substance Abuse Treatment Services
NY: New York
OH: Ohio
PA: Pennsylvania
PWID: People who inject drugs
SAMHSA: Substance Abuse and Mental Health Service Administration
TEDS: Treatment Episode Data Set
UFDS: Uniform Facility Data Set
US: United States
VA: Virginia

## Footnotes

1 In TEDS, non-injectors are defined as clients who use illicit drugs via other routes of administration than injection, such as oral, inhalation, or smoked.
